# Supplementary material for: The effects of social feedback on private opinions. Empirical evidence from the laboratory
Source: PLoS One. 2022 Oct 5;17(10):e0274903. doi: 10.1371/journal.pone.0274903 (PMC9534395; doi:10.1371/journal.pone.0274903)
Supplement: S1 Table — List of all social feedback statements used during the laboratory treatment process. (PDF) [file pone.0274903.s001.pdf]

The Effects of Social Feedback on Private Opinions.
Empirical Evidence from the Laboratory.

List of the Social Feedback Statements

Stephanie Jütersonke, Marcel Sarközi*

September 13, 2022

* marcel.sarkoezi@posteo.de

German (original)

Category	Social Feedback Statement	
very positive	1	Zum Glück gibt es noch vernünftige Menschen! Ich sehe das ganz genauso!
	2	Sehr schön, das ist eigentlich die einzig akzeptable Einstellung zu dem Thema!
	3	Ich bin sehr froh, dass ich nicht der einzige Mensch bin, der sich Gedanken macht und so denkt!
	4	Danke! Wenn mehr Leute so denken würden, wäre die Welt ein besserer Ort!
	5	Ich bin absolut der gleichen Meinung! Als denkender und fühlender Mensch muss man so antworten!
rather positive	6	Ähnlich würde ich es auch sehen.
	7	Das kann ich prinzipiell nur unterstützen.
	8	Eine grundsätzlich vernünftige Einstellung.
	9	Da kann man nichts dagegen sagen. Ich sehe es eigentlich genauso.
	10	Nach kurzer Überlegung würde ich mich dieser Meinung anschließen.
rather negative	11	Da würde ich nicht mitgehen. Es scheint, als ginge da das Denken tendenziell in die falsche Richtung.
	12	Das ist eine unerwartete, wenig unterstützenswerte Einstellung.
	13	Naja, also mir kommt diese Meinung etwas unüberlegt vor. Ich würde da nicht mitgehen.
	14	Puh, also das sehe ich anders. Ich finde, dass da nochmal drüber nachgedacht werden sollte.
	15	Ich weiß nicht, da hätte ich jetzt schon irgendwie eine andere Einstellung erwartet.
very negative	16	Ich frage mich ernsthaft, wie man heutzutage noch so eine Meinung vertreten kann!
	17	Au weja, wenn jeder so eine Einstellung hätte, dann wäre echt alles verloren!
	18	Autsch, das tut weh! Wie kommt man dazu, so über das Thema zu denken?
	19	So eine Meinung verstößt eigentlich gegen jede gesellschaftlich akzeptable Vorstellung vom Guten und Richtigen!
	20	Furchtbare Einstellung! Lehne ich entschieden ab!

English (translation)

Category	Social Feedback Statement	
very positive	1	Fortunately some people are still reasonable. I am of the exact same opinion!
	2	Very well, that is actually the only acceptable stand on this issue!
	3	I am so glad that I am not the only person who makes up their own mind and thinks this way!
	4	Thank you! If more people thought this way, the world would be a better place!
	5	I totally agree! There is no other way to respond for a thinking and feeling human being!
rather positive	6	I tend to agree.
	7	In principle, I agree.
	8	A generally reasonable attitude.
	9	There is no arguing this. In principle, I agree.
	10	After reconsidering it briefly, I agree.
rather negative	11	I do not agree. It seems that this kind of reasoning is leading in the wrong direction.
	12	This attitude is unexpected and hardly worth supporting.
	13	Well, this opinion seems a little inconsiderate to me. I would not go along with it.
	14	Phew, well I do not agree with this. I think that this needs to be given more thought.
	15	I don’t know, I kind of expected a different attitude.
very negative	16	Honestly, I find it hard to understand how anyone nowadays could express this kind of opinion!
	17	Whew, if everyone shared this kind of attitude, all would be lost!
	18	Ouch, that hurts! How could anyone start thinking this way about the topic?
	19	This kind of opinion contradicts every socially accepted notion of what is right and good!
	20	Horrible attitude! I strictly object!
