# Supplementary material for: The effects of social feedback on private opinions. Empirical evidence from the laboratory
Source: PLoS One. 2022 Oct 5;17(10):e0274903. doi: 10.1371/journal.pone.0274903 (PMC9534395; doi:10.1371/journal.pone.0274903)
Supplement: S1 Appendix — Explanations of all variables in the dataset. (PDF) [file pone.0274903.s002.pdf]

The Effects of Social Feedback on Private Opinions.
Empirical Evidence from the Laboratory.

Variable Report

Stephanie Jütersonke, Marcel Sarközi*

September 13, 2022

* marcel.sarkoezi@posteo.de

Overview and Frequencies of Variables

dataset

Study identifier

Alphanumerical string that identifies the data set.

dataset					
**Values**	**Label**	**Missing**	**Freq.**	**Percent**	**Valid Percent**
ODSF2018 - ODYCCEUS Social Feedback 2018			501	100.00	100.00
	Sum		501	100.00	100.00
	Valid Cases		501		

t1_age

T1 – Age

Participants age in years. The variable was measured during the first online survey (t_1).

t1_age					
**Values**	**Label**	**Missing**	**Freq.**	**Percent**	**Valid Percent**
valid values			445	88.82	100.00
not answered		M	56	11.18	
	Sum		501	100.00	100.00
	Valid Cases		445		

t1_age (valid values only)					
**Mean**	**Median**	**SD**	**Min**	**Max**	*N*
27.94	26	8.13	19	78	445

t1_bfi_ag

T1 – Agreeableness (BFI-10)

BFI-10 scale for the personality dimension *agreeableness*. The BFI-10 variables were measured during the first online survey (t_1).

t1_bfi_ag					
**Values**	**Label**	**Missing**	**Freq.**	**Percent**	**Valid Percent**
1			3	0.60	0.67
1.5			8	1.60	1.79
2			50	9.98	11.21
2.5			67	13.37	15.02
3			121	24.15	27.13
3.5			109	21.76	24.44
4			52	10.38	11.66
4.5			30	5.99	6.73
5			6	1.20	1.35
not answered		M	55	10.98	
	Sum		501	100.00	100.00
	Valid Cases		446		

t1_bfi_ag (valid values only)					
**Mean**	**Median**	**SD**	**Min**	**Max**	*N*
3.14	3	0.76	1	5	446

Rammstedt, Beatrice and Oliver P. John. 2017. “Measuring personality in one minute or less: A 10-item short version of the Big Five Inventory in English and German.” *Journal of Research in Personality Methods* 41, No 1:203-212.

t1_bfi_co

T1 – Conscientiousness (BFI-10)

BFI-10 scale for the personality dimension *conscientiousness*. The BFI-10 variables were measured during the first online survey (t_1).

t1_bfi_co					
**Values**	**Label**	**Missing**	**Freq.**	**Percent**	**Valid Percent**
1			0	0.00	0.00
1.5			5	1.00	1.12
2			16	3.19	3.59
2.5			37	7.39	8.30
3			88	17.56	19.73
3.5			111	22.16	24.89
4			83	16.57	18.61
4.5			80	15.97	17.94
5			26	5.19	5.83
not answered		M	55	10.98	
	Sum		501	100.00	100.00
	Valid Cases		446		

t1_bfi_co (valid values only)					
**Mean**	**Median**	**SD**	**Min**	**Max**	***N***
3.60	3.5	0.78	1.5	5	446

Rammstedt, Beatrice and Oliver P. John. 2017. “Measuring personality in one minute or less: A 10-item short version of the Big Five Inventory in English and German.” *Journal of Research in Personality Methods* 41, No 1:203-212.

t1_bfi_ex

T1 – Extraversion (BFI-10)

BFI-10 scale for the personality dimension *extraversion*. The BFI-10 variables were measured during the first online survey (t_1).

t1_bfi_ex					
**Values**	**Label**	**Missing**	**Freq.**	**Percent**	**Valid Percent**
1			8	1.60	1.79
1.5			18	3.59	4.04
2			56	11.18	12.56
2.5			54	10.78	12.11
3			68	13.57	15.25
3.5			71	14.17	15.92
4			112	22.36	25.11
4.5			40	7.98	8.97
5			19	3.79	4.26
not answered		M	55	10.98	
	Sum		501	100.00	100.00
	Valid Cases		446		

t1_bfi_ex (valid values only)					
**Mean**	**Median**	**SD**	**Min**	**Max**	***N***
3.27	3.5	0.95	1	5	446

Rammstedt, Beatrice and Oliver P. John. 2017. "Measuring personality in one minute or less: A 10-item short version of the Big Five Inventory in English and German." *Journal of Research in Personality Methods* 41, No 1:203-212.

t1_bfi_ne

T1 – Neuroticism (BFI 10)

BFI-10 scale for the personality dimension *neuroticism*. The BFI-10 variables were measured during the first online survey (t_1).

t1_bfi_ne					
**Values**	**Label**	**Missing**	**Freq.**	**Percent**	**Valid Percent**
1			8	1.60	1.79
1.5			23	4.59	5.16
2			78	15.57	17.49
2.5			74	14.77	16.59
3			80	15.97	17.94
3.5			61	12.18	13.68
4			74	14.77	16.59
4.5			38	7.58	8.52
5			10	2.00	2.24
not answered		M	55	10.98	
	Sum		501	100.00	100.00
	Valid Cases		446		

t1_bfi_ne (valid values only)					
**Mean**	**Median**	**SD**	**Min**	**Max**	*N*
3.04	3	0.94	1	5	446

Rammstedt, Beatrice and Oliver P. John. 2017. “Measuring personality in one minute or less: A 10-item short version of the Big Five Inventory in English and German.” *Journal of Research in Personality Methods* 41, No 1:203-212.

t1_bfi_op

T1 – Openness (BFI-10)

BFI-10 scale for the personality dimension *openness*. The BFI-10 variables were measured during the first online survey (t_1).

t1_bfi_op					
**Values**	**Label**	**Missing**	**Freq.**	**Percent**	**Valid Percent**
1			0	0.00	0.00
1.5			6	1.20	1.35
2			20	3.99	4.48
2.5			32	6.39	7.17
3			52	10.38	11.66
3.5			48	9.58	10.76
4			106	21.16	23.77
4.5			102	20.36	22.87
5			80	15.97	17.94
not answered		M	55	10.98	
	Sum		501	100.00	100.00
	Valid Cases		446		

t1_bfi_op (valid values only)					
**Mean**	**Median**	**SD**	**Min**	**Max**	***N***
3.90	4	0.89	1.5	5	446

Rammstedt, Beatrice and Oliver P. John. 2017. "Measuring personality in one minute or less: A 10-item short version of the Big Five Inventory in English and German." *Journal of Research in Personality Methods* 41, No 1:203-212.

t1_care

T1 – Carefulness in completing the questionnaire

Self-report on how carefully the questionnaire was completed. The variable was measured during the first online survey (t_1).

Original wording (German): "Bitte teilen Sie uns mit, wie sorgfältig Sie diesen Fragebogen ausgefüllt haben."

t1_care					
**Values**	**Label**	**Missing**	**Freq.**	**Percent**	**Valid Percent**
1	not carefully at all		0	0	0
2			0	0	0
3			0	0	0
4			3	0.60	0.68
5			28	5.59	6.31
6			176	35.13	39.64
7	very carefully		237	47.31	53.38
not answered		M	56	11.18	
.a		M	1	0.20	
	Sum		501	100.00	100.00
	Valid Cases		444		

t1_care (valid values only)					
**Mean**	**Median**	**SD**	**Min**	**Max**	N
6.46	7	0.64	4	7	444

t1_case

T1 – Interview number

Participant's interview number. System variable that was automatically generated by SoSci Survey (t_1).

t1_case					
**Values**	**Label**	**Missing**	**Freq.**	**Percent**	**Valid Percent**
valid values			455	90.82	100.00
not available		M	46	9.18	
	Sum		501	100.00	100.00
	Valid Cases		455		

t1_date

T1 – Day of the month on which the interview was started

Day in October 2018 on which the online survey was started. System variable that was automatically generated by SoSci Survey (t_1).

t1_date					
**Values**	**Label**	**Missing**	**Freq.**	**Percent**	**Valid Percent**
10			279	55.69	61.32
11			115	22.95	25.27
12			32	6.39	7.03
13			12	2.40	2.64
14			17	3.39	3.74
not available		M	46	9.18	
	Sum		501	100.00	100.00
	Valid Cases		455		

t1_job

T1 – Job

Variable reporting the occupation of participants. The variable was measured during the first online survey (t_1).

t1_job					
**Values**	**Label**	**Missing**	**Freq.**	**Percent**	**Valid Percent**
1	still at school		0	0.00	0.00
2	in apprenticeship		8	1.60	1.80
3	student		305	60.88	68.54
4	worker		5	1.00	1.12
5	office worker		79	15.77	17.75
6	official		4	0.80	0.90
7	self-employed		20	3.99	4.49
8	unemployed/job seeking		9	1.80	2.02
9	miscellaneous		15	2.99	3.37
not answered		M	56	11.18	
	Sum		501	100.00	100.00
	Valid Cases		445		

t1_lastdata

T1 – Time when the dataset was last changed

System variable that was automatically generated by SoSci Survey (t_1).

t1_lastdata					
**Values**	**Label**	**Missing**	**Freq.**	**Percent**	**Valid Percent**
10 Oct 18			274	54.69	60.22
11 Oct 18			118	23.55	25.93
12 Oct 18			33	6.59	7.25
13 Oct 18			13	2.59	2.86
14 Oct 18			17	3.39	3.74
not available		M	46	9.18	
	Sum		501	100.00	100.00
	Valid Cases		455		

t1_lastpage

T1 – Page the participants last edited

System variable that was automatically generated by SoSci Survey (t_1).

t1_lastpage					
**Values**	**Label**	**Missing**	**Freq.**	**Percent**	**Valid Percent**
2			2	0.40	0.44
3			2	0.40	0.44
4			2	0.40	0.44
7			2	0.40	0.44
11			1	0.20	0.22
13			1	0.20	0.22
17			15	2.99	3.30
18			430	85.83	94.51
not answered		M	46	9.18	
	Sum		501	100.00	100.00
	Valid Cases		455		

t1_quest

T1 – Questionnaire version

System variable that was automatically generated by SoSci Survey (t_1). It indicates which questionnaire was presented to the participants.

t1_quest					
**Values**	**Label**	**Missing**	**Freq.**	**Percent**	**Valid Percent**
0	control group		98	19.56	21.54
1	treatment group(s)		357	71.26	78.46
not available		M	46	9.18	
	Sum		501	100.00	100.00
	Valid Cases		455		

t1_sex

T1 – Sex

Self-reported sex. The variable was measured during the first online survey (t_1).

t1_sex					
**Values**	**Label**	**Missing**	**Freq.**	**Percent**	**Valid Percent**
0	female		316	63.07	71.01
1	male		126	25.15	28.31
2	other		3	0.60	0.67
not answered		M	56	11.18	
	Sum		501	100.00	100.00
	Valid Cases		445		

t1_sp

T1 – Speciesism Index

Index variable measuring participants' overall score on the Speciesism Scale. The variables involved in the formation were measured on a scale from 0 to 100 during the first online survey (t_1).

t1_sp					
**Values**	**Label**	**Missing**	**Freq.**	**Percent**	**Valid Percent**
valid values			414	82.63	100.00
not answered		M	87	17.37	
	Sum		501	100.00	100.00
	Valid Cases		414		

t1_sp (valid values only)					
**Mean**	**Median**	**SD**	**Min**	**Max**	***N***
37.62	37.59	17.85	0	87.78	414

Windrich, Ivo. 2017. "Zur Messung speziesistischer Einstellungen". Leipzig, *Arbeitsbericht des Instituts für Soziologie*, No. 72.

t1_sp01

T1 – Speciesism: Ich finde es in Ordnung, dass Tiere für die Fleischproduktion getötet werden.

English version: "I agree with the killing of animals for meat production.", own translation.

Speciesism Scale item. The variable was measured on a scale from 0 to 100 during the first online survey (t_1).

t1_sp01					
**Values**	**Label**	**Missing**	**Freq.**	**Percent**	**Valid Percent**
valid values			444	88.62	100.00
I don't know		M	6	1.20	
not answered		M	51	10.18	
	Sum		501	100.00	100.00
	Valid Cases		444		

t1_sp01 (valid values only)					
**Mean**	**Median**	**SD**	**Min**	**Max**	*N*
48.35	50.5	34.11	0	100	444

Windrich, Ivo. 2017. "Zur Messung speziesistischer Einstellungen". Leipzig, *Arbeitsbericht des Instituts für Soziologie*, No. 72.

t1_sp02

T1 – Speciesism: Tiere sind zum Nutzen des Menschen da.

English version: “Animals exist for the benefit of humans.”, own translation.

Speciesism Scale item. The variable was measured on a scale from 0 to 100 during the first online survey (t_1).

t1_sp02					
**Values**	**Label**	**Missing**	**Freq.**	**Percent**	**Valid Percent**
valid values			439	87.62	100.00
I don't know		M	11	2.20	
not answered		M	51	10.18	
	Sum		501	100.00	100.00
	Valid Cases		439		

t1_sp02 (valid values only)					
**Mean**	**Median**	**SD**	**Min**	**Max**	*N*
30.80	25	27.43	0	100	439

Windrich, Ivo. 2017. “Zur Messung speziesistischer Einstellungen”. Leipzig, *Arbeitsbericht des Instituts für Soziologie*, No. 72.

t1_sp03

T1–Speciesism: Ich lehne Tierversuche ab (reverse wording).

English version: “I oppose animal testing.”, own translation.

Speciesism Scale item. The variable was measured on a scale from 0 to 100 during the first online survey (t_1).

t1_sp03					
**Values**	**Label**	**Missing**	**Freq.**	**Percent**	**Valid Percent**
valid values			449	89.62	100.00
I don't know		M	1	0.20	
not answered		M	51	10.18	
	Sum		501	100.00	100.00
	Valid Cases		449		

t1_sp03 (valid values only)					
**Mean**	**Median**	**SD**	**Min**	**Max**	***N***
33.58	22	33.58	0	100	449

Remarks: The item was reverse worded and has been recoded accordingly.

Windrich, Ivo. 2017. “Zur Messung speziesistischer Einstellungen”. Leipzig, *Arbeitsbericht des Instituts für Soziologie*, No. 72.

t1_sp04

T1 – Speciesism: Nur Menschen haben ein Bewusstsein, Tiere haben kein Bewusstsein.

English version: "Only human beings have consciousness, animals do not have consciousness.", own translation.

Speciesism Scale item. The variable was measured on a scale from 0 to 100 during the first online survey (t_1).

t1_sp04					
**Values**	**Label**	**Missing**	**Freq.**	**Percent**	**Valid Percent**
valid values			435	86.83	100.00
I don't know		M	15	2.99	
not answered		M	51	10.18	
	Sum		501	100.00	100.00
	Valid Cases		435		

t1_sp04 (valid values only)					
**Mean**	**Median**	**SD**	**Min**	**Max**	N
17.08	5	24.51	0	100	435

Windrich, Ivo. 2017. "Zur Messung speziesistischer Einstellungen". Leipzig, *Arbeitsbericht des Instituts für Soziologie*, No. 72.

t1_sp05

T1 – Speciesism: Die Vernunft trennt den Menschen vom Tier.

English version: “Reason separates human beings from animals.”, own translation.

Speciesism Scale item. The variable was measured on a scale from 0 to 100 during the first online survey (t_1).

t1_sp05					
**Values**	**Label**	**Missing**	**Freq.**	**Percent**	**Valid Percent**
valid values			445	88.82	100.00
I don't know		M	5	1.00	
not answered		M	51	10.18	
	Sum		501	100.00	100.00
	Valid Cases		445		

t1_sp05 (valid values only)					
**Mean**	**Median**	**SD**	**Min**	**Max**	***N***
49.20	49	32.18	0	100	445

Windrich, Ivo. 2017. “Zur Messung speziesistischer Einstellungen”. Leipzig, *Arbeitsbericht des Instituts für Soziologie*, No. 72.

t1_sp06

T1 – Speciesism: Die Massentierhaltung ist nicht mit den Moralvorstellungen unserer Gesellschaft vereinbar (reverse wording)

English version: "Intensive livestock farming is incompatible with our society's code of ethics.", own translation.

Attention: This variable is not part of the original Speciesism Scale.

The variable was measured on a scale from 0 to 100 during the first online survey (t_1).

t1_sp06					
**Values**	**Label**	**Missing**	**Freq.**	**Percent**	**Valid Percent**
valid values			450	89.82	100.00
not answered		M	51	10.18	
	Sum		501	100.00	100.00
	Valid Cases		450		

t1_sp06 (valid values only)					
**Mean**	**Median**	**SD**	**Min**	**Max**	***N***
24.71	11.5	30.42	0	100	450

Remarks: The item was reverse worded and has been recoded accordingly.

Windrich, Ivo. 2017. "Zur Messung speziesistischer Einstellungen". Leipzig, *Arbeitsbericht des Instituts für Soziologie*, No. 72.

t1_sp07

T1 – Speciesism: Die Menschen sollten Tiere als fühlende Mitlebewesen respektieren (reverse wording).

English version: "Humans should respect animals as sentient co-beings.", own translation.

Speciesism Scale item. The variable was measured on a scale from 0 to 100 during the first online survey (t_1).

t1_sp07					
**Values**	**Label**	**Missing**	**Freq.**	**Percent**	**Valid Percent**
valid values			449	89.62	100.00
not answered		M	52	10.38	
	Sum		501	100.00	100.00
	Valid Cases		449		

t1_sp07 (valid values only)					
**Mean**	**Median**	**SD**	**Min**	**Max**	N
15.55	7	20.01	0	100	449

Remarks: The item was reverse worded and has been recoded accordingly.

Windrich, Ivo. 2017. "Zur Messung speziesistischer Einstellungen". Leipzig, *Arbeitsbericht des Instituts für Soziologie*, No. 72.

t1_sp08

T1 – Speciesism: Tiere sind ihrem Trieb vollkommen unterworfen.

English version: "Animals are completely subdued to their instincts.", own translation.

Speciesism Scale item. The variable was measured on a scale from 0 to 100 during the first online survey (t_1).

t1_sp08					
**Values**	**Label**	**Missing**	**Freq.**	**Percent**	**Valid Percent**
valid values			448	89.42	100.00
I don't know		M	1	0.20	
not answered		M	52	10.38	
	Sum		501	100.00	100.00
	Valid Cases		448		

t1_sp08 (valid values only)					
**Mean**	**Median**	**SD**	**Min**	**Max**	***N***
51.61	50	26.16	0	100	448

Windrich, Ivo. 2017. "Zur Messung speziesistischer Einstellungen". Leipzig, *Arbeitsbericht des Instituts für Soziologie*, No. 72.

t1_sp09

T1 – Speciesism (target item): Tiere zu töten und zu essen gehört einfach zum Wesen des Menschen.

English version: "The killing and eating of animals is part of human nature.", own translation.

Speciesism Scale item. The variable was measured on a scale from 0 to 100 during the first online survey (t_1).

t1_sp09					
**Values**	**Label**	**Missing**	**Freq.**	**Percent**	**Valid Percent**
valid values			442	88.22	100.00
I don't know		M	7	1.40	
not answered		M	52	10.38	
	Sum		501	100.00	100.00
	Valid Cases		442		

t1_sp09 (valid values only)					
**Mean**	**Median**	**SD**	**Min**	**Max**	*N*
45.77	48	32.77	0	100	442

Windrich, Ivo. 2017. "Zur Messung speziesistischer Einstellungen". Leipzig, *Arbeitsbericht des Instituts für Soziologie*, No. 72.

t1_sp10

T1 – Speciesism: Ich betrachte Tiere als gleichwertig zum Menschen (reverse wording).

English version: “I consider animals to be of equal value as human beings.”, own translation.

Speciesism Scale item. The variable was measured on a scale from 0 to 100 during the first online survey (t_1).

t1_sp10					
**Values**	**Label**	**Missing**	**Freq.**	**Percent**	**Valid Percent**
valid values			449	89.62	100.00
not answered		M	52	10.38	
	Sum		501	100.00	100.00
	Valid Cases		449		

t1_sp10 (valid values only)					
**Mean**	**Median**	**SD**	**Min**	**Max**	***N***
51.24	52	31.38	0	100	449

Windrich, Ivo. 2017. “Zur Messung speziesistischer Einstellungen”. Leipzig, *Arbeitsbericht des Instituts für Soziologie*, No. 72.

Remarks: The item was reverse worded and has been recoded accordingly.

t1_sp11

T1 – Speciesism: Der Mensch soll bestimmen, was mit Tieren passiert.

English version: "Human beings should decide over animals.", own translation.

Speciesism Scale item. The variable was measured on a scale from 0 to 100 during the first online survey (t_1).

t1_sp11					
**Values**	**Label**	**Missing**	**Freq.**	**Percent**	**Valid Percent**
valid values			439	87.62	100.00
I don't know		M	10	2.00	
not answered		M	52	10.38	
	Sum		501	100.00	100.00
	Valid Cases		439		

t1_sp11 (valid values only)					
**Mean**	**Median**	**SD**	**Min**	**Max**	N
30.57	24	26.02	0	100	439

Windrich, Ivo. 2017. "Zur Messung speziesistischer Einstellungen". Leipzig, *Arbeitsbericht des Instituts für Soziologie*, No. 72.

t1_sp12

T1 – Speciesism: Fische können Schmerzen empfinden (reverse wording).

English version: “Fish can feel pain.”, own translation.

Speciesism Scale item. The variable was measured on a scale from 0 to 100 during the first online survey (t_1).

t1_sp12					
**Values**	**Label**	**Missing**	**Freq.**	**Percent**	**Valid Percent**
valid values			447	89.22	100.00
I don't know		M	2	0.40	
not answered		M	52	10.38	
	Sum		501	100.00	100.00
	Valid Cases		447		

t1_sp12 (valid values only)					
**Mean**	**Median**	**SD**	**Min**	**Max**	***N***
24.24	19	24.94	0	100	447

Remarks: The item was reverse worded and has been recoded accordingly.

Windrich, Ivo. 2017. “Zur Messung speziesistischer Einstellungen”. Leipzig, *Arbeitsbericht des Instituts für Soziologie*, No. 72.

t1_sp13

T1 – Speciesism: Meiner Meinung nach steht der Mensch über dem Tier.

English version: "In my opinion human beings are superior to animals.", own translation.

Speciesism Scale item. The variable was measured on a scale from 0 to 100 during the first online survey (t_1).

t1_sp13					
**Values**	**Label**	**Missing**	**Freq.**	**Percent**	**Valid Percent**
valid values			443	88.42	100.00
I don't know		M	6	1.20	
not answered		M	52	10.38	
	Sum		501	100.00	100.00
	Valid Cases		443		

t1_sp13 (valid values only)					
**Mean**	**Median**	**SD**	**Min**	**Max**	***N***
46.03	49	33.67	0	100	443

Windrich, Ivo. 2017. "Zur Messung speziesistischer Einstellungen". Leipzig, *Arbeitsbericht des Instituts für Soziologie*, No. 72.

t1_sp14

T1 – Speciesism: Nutztiere sind nun einmal dazu da geschlachtet zu werden.

English version: “Livestock exists to be slaughtered.”, own translation.

Speciesism Scale item. The variable was measured on a scale from 0 to 100 during the first online survey (t_1).

t1_sp14					
**Values**	**Label**	**Missing**	**Freq.**	**Percent**	**Valid Percent**
valid values			443	88.42	100.00
I don't know		M	6	1.20	
not answered		M	52	10.38	
	Sum		501	100.00	100.00
	Valid Cases		443		

t1_sp14 (valid values only)					
**Mean**	**Median**	**SD**	**Min**	**Max**	***N***
39.06	33	32.83	0	100	443

Windrich, Ivo. 2017. “Zur Messung speziesistischer Einstellungen”. Leipzig, *Arbeitsbericht des Instituts für Soziologie*, No. 72.

t1_sp15

T1 – Speciesism: Ich rege mich manchmal auf, wenn ich wilde Tiere in Käfigen im Zoo sehe (reverse wording).

English version: "I sometimes get furious upon viewing wild animals caged in zoos.", own translation.

Speciesism Scale item. The variable was measured on a scale from 0 to 100 during the first online survey (t_1).

t1_sp15					
**Values**	**Label**	**Missing**	**Freq.**	**Percent**	**Valid Percent**
valid values			449	89.62	100.00
not answered		M	52	10,38	
	Sum		501	100.00	100.00
	Valid Cases		449		

t1_sp15 (valid values only)					
**Mean**	**Median**	**SD**	**Min**	**Max**	***N***
31.38	24	29.43	0	100	449

Remarks: The item was reverse worded and has been recoded accordingly.

Windrich, Ivo. 2017. "Zur Messung speziesistischer Einstellungen". Leipzig, *Arbeitsbericht des Instituts für Soziologie*, No. 72.

t1_sp16

T1 – Speciesism: Manche Tiere sind Nahrungsmittel.

English version: "Some animals are food items.", own translation.

Speciesism Scale item. The variable was measured on a scale from 0 to 100 during the first online survey (t_1).

t1_sp16					
**Values**	**Label**	**Missing**	**Freq.**	**Percent**	**Valid Percent**
valid values			442	88.22	100.00
I don't know		M	7	1.40	
not answered		M	52	10.38	
	Sum		501	100.00	100.00
	Valid Cases		442		

t1_sp16 (valid values only)

**Mean**	**Median**	**SD**	**Min**	**Max**	N
45.38	48	33.94	0	100	442

Windrich, Ivo. 2017. "Zur Messung speziesistischer Einstellungen". Leipzig, *Arbeitsbericht des Instituts für Soziologie*, No. 72.

t1_sp17

T1 – Speciesism: Manche Tiere können Leid, Freude und Trauer empfinden (reverse wording).

English version: “Some animals are capable of feeling pain, joy and grief.”, own translation.

Speciesism Scale item. The variable was measured on a scale from 0 to 100 during the first online survey (t_1).

t1_sp17					
**Values**	**Label**	**Missing**	**Freq.**	**Percent**	**Valid Percent**
valid values			447	89.22	100.00
I don't know		M	2	0.40	
not answered		M	52	10.38	
	Sum		501	100.00	100.00
	Valid Cases		474		

t1_sp17 (valid values only)					
**Mean**	**Median**	**SD**	**Min**	**Max**	N
9.76	1	16.64	0	100	447

Remarks: The item was reverse worded and has been recoded accordingly.

Windrich, Ivo. 2017. “Zur Messung speziesistischer Einstellungen”. Leipzig, *Arbeitsbericht des Instituts für Soziologie*, No. 72.

t1_sp18

T1 – Speciesism: Wenn Kühe ein glückliches Leben haben, dann ist es in Ordnung ihre Milch zu nehmen.

English version: "If cows live a happy life it is acceptable to take their milk.", own translation.

Speciesism Scale item. The variable was measured on a scale from 0 to 100 during the first online survey (t_1).

t1_sp18					
**Values**	**Label**	**Missing**	**Freq.**	**Percent**	**Valid Percent**
valid values			448	89.42	100.00
I don't know		M	1	0.20	
not answered		M	52	10.38	
	Sum		501	100.00	100.00
	Valid Cases		448		

t1_sp18 (valid values only)					
**Mean**	**Median**	**SD**	**Min**	**Max**	***N***
70.48	78	29.21	0	100	448

Windrich, Ivo. 2017. "Zur Messung speziesistischer Einstellungen". Leipzig, *Arbeitsbericht des Instituts für Soziologie*, No. 72.

t1_sp19

T1 – Speciesism: Der Körper eines Schweines gehört ausschließlich dem Schwein (reverse wording).

English version: “A pig’s body belongs exclusively to the pig.”, own translation.

Speciesism Scale item. The variable was measured on a scale from 0 to 100 during the first online survey (t_1).

t1_sp19					
**Values**	**Label**	**Missing**	**Freq.**	**Percent**	**Valid Percent**
valid values			447	89.22	100.00
I don't know		M	2	0.40	
not answered		M	52	10.38	
	Sum		501	100.00	100.00
	Valid Cases		447		

t1_sp19 (valid values only)					
**Mean**	**Median**	**SD**	**Min**	**Max**	N
38.38	40	29.97	0	100	447

Remarks: The item was reverse worded and has been recoded accordingly.

Windrich, Ivo. 2017. “Zur Messung speziesistischer Einstellungen”. Leipzig, *Arbeitsbericht des Instituts für Soziologie*, No. 72.

t1_sp20

T1 – Speciesism: Tiere sollten ihr eigenes Leben leben dürfen (reverse wording).

English version: "Animals should be allowed to live their own life.", own translation.

Speciesism Scale item. The variable was measured on a scale from 0 to 100 during the first online survey (t_1).

t1_sp20					
**Values**	**Label**	**Missing**	**Freq.**	**Percent**	**Valid Percent**
valid values			447	89.22	100.00
I don't know		M	2	0.40	
not answered		M	52	10.38	
	Sum		501	100.00	100.00
	Valid Cases		447		

t1_sp20 (valid values only)					
**Mean**	**Median**	**SD**	**Min**	**Max**	***N***
29.75	27	24.38	0	100	447

Remarks: The item was reverse worded and has been recoded accordingly.

Windrich, Ivo. 2017. "Zur Messung speziesistischer Einstellungen". Leipzig, *Arbeitsbericht des Instituts für Soziologie*, No. 72.

t1_sp21

T1 – Speciesism: Das Nutztiere getötet werden, ist kein besonderes Problem, es sind ja bloß Tiere.

English version: "Killing of livestock is not problematic, it's just animals.", own translation.

Speciesism Scale item. The variable was measured on a scale from 0 to 100 during the first online survey (t_1).

t1_sp21					
**Values**	**Label**	**Missing**	**Freq.**	**Percent**	**Valid Percent**
valid values			437	87.23	100.00
I don't know		M	12	2.40	
not answered		M	52	10.38	
	Sum		501	100.00	100.00
	Valid Cases		437		

t1_sp21 (valid values only)					
**Mean**	**Median**	**SD**	**Min**	**Max**	*N*
21.48	11	24.19	0	100	437

Windrich, Ivo. 2017. "Zur Messung speziesistischer Einstellungen". Leipzig, *Arbeitsbericht des Instituts für Soziologie*, No. 72.

t1_sp22

T1 – Speciesism: Ich finde, dass das Schlachten von Tieren ein Ende finden sollte (reverse wording).

English version: “I think there should be an end to slaughtering animals.”, own translation.

Speciesism Scale item. The variable was measured on a scale from 0 to 100 during the first online survey (t_1).

t1_sp22					
**Values**	**Label**	**Missing**	**Freq.**	**Percent**	**Valid Percent**
valid values			446	89.02	100.00
I don't know		M	3	0.60	
not answered		M	52	10.38	
	Sum		501	100.00	100.00
	Valid Cases		446		

t1_sp22 (valid values only)					
**Mean**	**Median**	**SD**	**Min**	**Max**	N
48.46	51	33.75	0	100	446

Remarks: The item was reverse worded and has been recoded accordingly.

Windrich, Ivo. 2017. “Zur Messung speziesistischer Einstellungen”. Leipzig, *Arbeitsbericht des Instituts für Soziologie*, No. 72.

t1_sp23

T1 – Speciesism: Wenn ich einen Tiertransporter sehe, dann macht mich das wütend oder traurig (reverse wording).

English version: “Whenever I see an animal transport I get angry or sad.”, own translation.

Speciesism Scale item. The variable was measured on a scale from 0 to 100 during the first online survey (t_1).

t1_sp23					
**Values**	**Label**	**Missing**	**Freq.**	**Percent**	**Valid Percent**
valid values			449	89.62	100.00
not answered		M	52	10.38	
	Sum		501	100.00	100.00
	Valid Cases		449		

t1_sp23 (valid values only)					
**Mean**	**Median**	**SD**	**Min**	**Max**	***N***
33.91	29	31.14	0	100	449

Remarks: The item was reverse worded and has been recoded accordingly.

Windrich, Ivo. 2017. “Zur Messung speziesistischer Einstellungen”. Leipzig, *Arbeitsbericht des Instituts für Soziologie*, No. 72.

t1_sp24

T1 – Speciesism: Der Schmerz eines Menschen zählt mehr als der Schmerz einer Maus.

English version: "The pain of a human being is of greater relevance than a mouse's pain.", own translation.

Speciesism Scale item. The variable was measured on a scale from 0 to 100 during the first online survey (t_1).

t1_sp24					
**Values**	**Label**	**Missing**	**Freq.**	**Percent**	**Valid Percent**
valid values			442	88.22	100.00
I don't know		M	7	1.40	
not answered		M	52	10.38	
	Sum		501	100.00	100.00
	Valid Cases		442		

t1_sp24 (valid values only)					
**Mean**	**Median**	**SD**	**Min**	**Max**	***N***
46.02	49	34.03	0	100	442

Remarks: The item was reverse worded and has been recoded accordingly.

Windrich, Ivo. 2017. "Zur Messung speziesistischer Einstellungen". Leipzig, *Arbeitsbericht des Instituts für Soziologie*, No. 72.

t1_started

T1 – Time at which the interview started.

System variable that was automatically generated by SoSci Survey (t_1).

t1_lastdata					
**Values**	**Label**	**Missing**	**Freq.**	**Percent**	**Valid Percent**
10 Oct 18			279	55.69	61.32
11 Oct 18			115	22.95	25.27
12 Oct 18			32	6.39	7.03
13 Oct 18			12	2.40	2.64
14 Oct 18			17	3.39	3.74
not available		M	46	9.18	
	Sum		501	100.00	100.00
	Valid Cases		455		

t1_time_sum

T1 – Total time spent (outliers excluded)

System variable that was automatically generated by SoSci Survey (t_1).

t1_time_sum					
**Mean**	**Median**	**SD**	**Min**	**Max**	*N*
649.45	621	219.13	33	1485	455

t2_date

T2 – Day of the month on which the session took place

Day in October 2018 on which the lab session took place. System variable that was automatically generated by z-Tree (t_2).

t2_date					
**Values**	**Label**	**Missing**	**Freq.**	**Percent**	**Valid Percent**
17			42	19.27	8.38
19			28	12.84	5.59
20			36	16.51	7.19
22			42	19.27	8.38
24			36	16.51	7.19
26			22	10.09	4.39
27			12	5.50	2.40
not available		M	283	56.49	
	Sum		501	100.00	100.00
	Valid Cases		218		

t2_doubt

T2 – Has the participant expressed any doubts about the alleged intention?

Variable indicating whether participants expressed doubts about the alleged interaction process in the comment section. The variable was collected during the laboratory sessions (t_2).

t2_doubt					
**Values**	**Label**	**Missing**	**Freq.**	**Percent**	**Valid Percent**
0	no		494	98.60	98.60
1	yes		7	1.40	1.40
	Sum		501	100.00	100.00
	Valid Cases		501		

t2_session

T2 – Laboratory session number

Laboratory session identification number. System variable that was automatically generated by z-Tree during the lab sessions (t_2).

t2_session					
**Values**	**Label**	**Missing**	**Freq.**	**Percent**	**Valid Percent**
1			13	2.59	5.96
2			14	2.79	6.42
3			15	2.99	6.88
4			13	2.59	5.96
5			15	2.99	6.88
6			15	2.99	6.88
7			11	2.20	5.05
8			10	2.00	4.59
9			12	2.40	5.50
10			15	2.99	6.88
11			15	2.99	6.88
12			11	2.20	5.05
13			16	3.19	7.34
14			9	1.80	4.13
15			8	1.60	3.67
16			14	2.79	6.42
17			6	1.20	2.75
18			6	1.20	2.75
.		M	283	56.49	
	Sum		501	100.00	100.00
	Valid Cases		218		

t2_sfb1

T2 – First social feedback statement (group member A, first line)

These made-up statements were randomly presented to participants as alleged group member A's feedback on their previously stated opinions during the laboratory session (t_2).

t2_sfb1					
**Values**	**Label** (German original)	**Missing**	**Freq.**	**Percent**	**Valid Percent**
1	Zum Glück gibt es noch vernünftige Menschen! Ich sehe das ganz genauso!		23	4.59	10.55
3	Ich bin sehr froh, dass ich nicht der einzige Mensch bin, der sich Gedanken macht und so denkt!		25	4.99	11.47
5	Ich bin absolut der gleichen Meinung! Als denkender und fühlender Mensch muss man so antworten!		14	2.79	6.42
7	Das kann ich prinzipiell nur unterstützen.		16	3.19	7.34
9	Da kann man nichts dagegen sagen. Ich sehe es eigentlich genauso.		25	4.99	11.47
11	Da würde ich nicht mitgehen. Es scheint, als ginge da das Denken tendenziell in die falsche Richtung.		21	4.19	9.63
13	Naja, also mir kommt diese Meinung etwas unüberlegt vor. Ich würde da nicht mitgehen.		24	4.79	11.01
15	Ich weiß nicht, da hätte ich jetzt schon irgendwie eine andere Einstellung erwartet.		23	4.59	10.55
17	Au weja, wenn jeder so eine Einstellung hätte, dann wäre echt alles verloren!		27	5.39	12.39
19	So eine Meinung verstößt eigentlich gegen jede gesellschaftlich akzeptable Vorstellung vom Guten und Richtigen!		20	3.99	9.17
.		M	283	56.49	
	Sum		501	100.00	100.00
	Valid Cases		218		

t2_sfb1					
**Values**	**Label** (English translation)	**Missing**	**Freq.**	**Percent**	**Valid Percent**
1	Fortunately some people are still reasonable. I am of the exact same opinion!		23	4.59	10.55
3	I am so glad that I am not the only person who makes up their own mind and thinks this way!		25	4.99	11.47
5	I totally agree! There is no other way to respond for a thinking and feeling human being!		14	2.79	6.42
7	In principle, I agree.		16	3.19	7.34
9	There is no arguing this. In principle, I agree.		25	4.99	11.47
11	I do not agree. It seems that this kind of reasoning is leading in the wrong direction.		21	4.19	9.63
13	Well, this opinion seems a little inconsiderate to me. I would not go along with it.		24	4.79	11.01
15	I don't know, I kind of expected a different attitude.		23	4.59	10.55
17	Whew, if everyone shared this kind of attitude, all would be lost!		27	5.39	12.39
19	This kind of opinion contradicts every socially accepted notion of what is right and good!		20	3.99	9.17
.		M	283	56.49	
	Sum		501	100.00	100.00
	Valid Cases		218		

t2_sfb2

T2 – Second social feedback statement (group member C, second line)

These made-up statements were randomly presented to participants as alleged group member C's feedback on their previously stated opinions during the laboratory session (t_2).

t2_sfb2					
**Values**	**Label**	**Missing**	**Freq.**	**Percent**	**Valid Percent**
2	Sehr schön, das ist eigentlich die einzig akzeptable Einstellung zu dem Thema!		18	3.59	8.26
4	Danke! Wenn mehr Leute so denken würden, wäre die Welt ein besserer Ort!		22	4.43	10.09
6	Ähnlich würde ich es auch sehen.		19	3.79	8.72
8	Eine grundsätzlich vernünftige Einstellung.		21	4.19	9.63
10	Nach kurzer Überlegung würde ich mich dieser Meinung anschließen.		21	4.19	9.63
12	Das ist eine unerwartete, wenig unterstützenswerte Einstellung.		25	4.99	11.47
14	Puh, also das sehe ich anders. Ich finde, dass da nochmal drüber nachgedacht werden sollte.		16	3.19	7.34
16	Ich frage mich ernsthaft, wie man heutzutage noch so eine Meinung vertreten kann!		36	7.19	16.51
18	Autsch, das tut weh! Wie kommt man dazu, so über das Thema zu denken?		19	3.79	8.72
20	Furchtbare Einstellung! Lehne ich entschieden ab!		21	4.19	9.63
.		M	283	56.49	
	Sum		501	100.00	100.00
	Valid Cases		218		

t2_sfb2					
**Values**	**Label** (English translation)	**Missing**	**Freq.**	**Percent**	**Valid Percent**
2	Very well, that is actually the only acceptable stand on this issue!		18	3.59	8.26
4	Thank you! If more people thought this way, the world would be a better place!		22	4.43	10.09
6	I tend to agree.		19	3.79	8.72
8	A generally reasonable attitude.		21	4.19	9.63
10	After reconsidering it briefly, I agree.		21	4.19	9.63
12	This attitude is unexpected and hardly worth supporting.		25	4.99	11.47
14	Phew, well I do not agree with this. I think that this needs to be given more thought.		16	3.19	7.34
16	Honestly, I find it hard to understand how anyone nowadays could express this kind of opinion!		36	7.19	16.51
18	Ouch, that hurts! How could anyone start thinking this way about the topic?		19	3.79	8.72
20	Horrible attitude! I strictly object!		21	4.19	9.63
.		M	283	56.49	
	Sum		501	100.00	100.00
	Valid Cases		218		

t2_subject

T2 – Participant number (within the laboratory session)

Participants identification number during the respective laboratory session. System variable that was automatically generated by z-Tree during the lab sessions (t_2).

t2_tg

T2 – Laboratory treatment group membership

Treatment condition the participants were randomly assigned to during the laboratory session (t_2).

t2_tg					
**Values**	**Label**	**Missing**	**Freq.**	**Percent**	**Valid Percent**
1	positive-positive		53	10.58	24.31
2	negative-negative		67	13.37	30.73
3	positive-negative		50	9.98	22.94
4	negative-positive		48	9.58	22.02
not answered		M	283	56.49	
	Sum		501	100.00	100.00
	Valid Cases		218		

t2_time

T2 – Time at which the laboratory session started

Time at which the laboratory session started. System variable that was automatically generated by z-Tree during the lab sessions (t_2).

t2_time					
**Values**	**Label**	**Missing**	**Freq.**	**Percent**	**Valid Percent**
181017_0835			13	2.59	5.96
181017_0935			14	2.79	6.42
181017_1835			15	2.99	6.88
181019_0838			13	2.59	5.96
181019_0937			15	2.99	6.88
181020_1228			15	2.99	6.88
181020_1336			11	2.20	5.05
181022_1434			10	2.00	4.59
181022_1459			12	2.40	5.50
181022_1545			15	2.99	6.88
181024_1902			15	2.99	6.88
181024_0837			11	2.20	5.05
181024_0942			16	3.19	7.34
181024_1954			9	1.80	4.13
181026_0834			8	1.60	3.67
181026_0930			14	2.79	6.42
181027_1232			6	1.20	2.75
181027_1333			6	1.20	2.75
not available		M	283	56.49	
	Sum		501	100.00	100.00
	Valid Cases		218		

t3_age

T3 – Age

Participants age in years. The variable was measured during the second online survey (t_3).

t3_age					
**Values**	**Label**	**Missing**	**Freq.**	**Percent**	**Valid Percent**
valid values			265	52.89	100.00
not answered		M	236	47.11	
	Sum		501	100.00	100.00
	Valid Cases		265		

t3_age (valid values only)					
**Mean**	**Median**	**SD**	**Min**	**Max**	*N*
27.40	26	7.61	19	78	265

t3_care

T1 – Carefulness in completing the questionnaire

Self-report on how carefully the questionnaire was completed. The variable was measured during the second online survey (t_3).

Original wording (German): "Bitte teilen Sie uns mit, wie sorgfältig Sie diesen Fragebogen ausgefüllt haben."

t3_care					
**Values**	**Label**	**Missing**	**Freq.**	**Percent**	**Valid Percent**
1	not carefully at all		0	0	0
2			0	0	0
3			0	0	0
4			4	0.80	1.52
5			30	5.99	11.36
6			105	20.96	39.77
7	very carefully		125	24.95	47.35
not answered		M	237	47.31	
	Sum		501	100.00	100.00
	Valid Cases		264		

t3_care (valid values only)					
**Mean**	**Median**	**SD**	**Min**	**Max**	N
6.33	6	0.74	4	7	264

t3_case

T3 – Interview number

Participants interview number. System variable that was automatically generated by SoSci Survey (t_3).

t3_case					
**Values**	**Label**	**Missing**	**Freq.**	**Percent**	**Valid Percent**
valid values			270	53.89	100.00
not available		M	231	46.11	
	Sum		501	100.00	100.00
	Valid Cases		455		

t3_date

T3 – Day of the month on which the interview was started

Day in October 2018 on which the online survey was started. Values 32 and 33 correspond to November 1 and November 2, 2018. System variable that was automatically generated by SoSci Survey (t_3).

t3_date					
**Values**	**Label**	**Missing**	**Freq.**	**Percent**	**Valid Percent**
24			95	18.96	35.19
25			36	7.19	13.33
26			14	2.79	5.19
27			59	11.78	21.85
28			29	5.79	10.74
29			15	2.99	5.56
30			11	2.20	4.07
31			7	1.40	2.59
32			2	0.40	0.74
33			2	0.40	0.74
not available		M	231	46.11	
	Sum		501	100.00	100.00
	Valid Cases		270		

t3_diet

T3 – Diet

Participants were asked to share their eating habits. The variable was measured during the second online survey (t_3).

t3_diet					
**Values**	**Label**	**Missing**	**Freq.**	**Percent**	**Valid Percent**
1	with meat		53	10.58	20.00
2	with little meat		115	22.95	43.40
3	organic meat only		14	2.79	5.28
4	with fish, but without meat		17	3.39	6.42
5	vegetarian		33	6.59	12.45
6	mostly vegan		15	2.99	5.66
7	vegan		8	1.60	3.02
8	other		10	2.00	3.77
not answered		M	236	47.11	
	Sum		501	100.00	100.00
	Valid Cases		265		

t3_eval01

T3 – Evaluation FS1: Zum Glück gibt es noch vernünftige Menschen! Ich sehe das ganz genauso!

Participants were asked to share how positive or negative they would perceive the feedback statement above if they received it in a social situation. The variable was measured during the second online survey (t_3).

English version: "Fortunately some people are still reasonable. I am of the exact same opinion!", own translation.

t3_eval01					
**Values**	**Label**	**Missing**	**Freq.**	**Percent**	**Valid Percent**
1	very negative		2	0.40	0.99
2	negative		6	1.20	2.97
3	neutral		17	3.39	8.42
4	positive		51	10.18	25.25
5	very positive		126	25.15	62.38
not answered		M	299	56.68	
	Sum		501	100.00	100.00
	Valid Cases		202		

t3_eval01					
**Mean**	**Median**	**SD**	**Min**	**Max**	N
–	5	–	1	5	202

t3_eval02

T3 – Sehr schön, das ist eigentlich die einzig akzeptable Einstellung zu dem Thema.

Participants were asked to share how positive or negative they would perceive the feedback statement above if they received it in a social situation. The variable was measured during the second online survey (t_3).

English version: "Very well, that is actually the only acceptable stand on this issue!", own translation.

Participants were asked to share their perception of the above given statement. The variable was measured during the second online survey (t_3).

t3_eval02					
**Values**	**Label**	**Missing**	**Freq.**	**Percent**	**Valid Percent**
1	very negative		1	0.20	0.50
2	negative		10	2.00	4.95
3	neutral		18	3.59	8.91
4	positive		56	11.18	27.72
5	very positive		117	23.35	57.92
not answered		M	299	59.68	
	Sum		501	100.00	100.00
	Valid Cases		202		

t3_eval02					
**Mean**	**Median**	**SD**	**Min**	**Max**	***N***
–	5	–	1	5	202

t3_eval03

T3 – Evaluation FS3: Ich bin sehr froh, dass ich nicht der einzige Mensch bin, der sich Gedanken macht und so denkt!

Participants were asked to share how positive or negative they would perceive the feedback statement above if they received it in a social situation. The variable was measured during the second online survey (t_3).

English version: "I am so glad that I am not the only person who makes up their own mind and thinks this way!", own translation.

Participants were asked to share their perception of the above given statement. The variable was measured during the second online survey (t_3).

t3_eval03					
**Values**	**Label**	**Missing**	**Freq.**	**Percent**	**Valid Percent**
1	very negative		1	0.20	0.50
2	negative		4	0.80	1.98
3	neutral		14	2.79	6.93
4	positive		89	17.76	44.06
5	very positive		94	18.76	46.53
not answered		M	299	59.68	
	Sum		501	100.00	100.00
	Valid Cases		202		

t3_eval03					
**Mean**	**Median**	**SD**	**Min**	**Max**	***N***
–	4	–	1	5	202

t3_eval04

T3 – Evaluation FS4: Danke! Wenn mehr Leute so denken würden, wäre die Welt ein besserer Ort.

Participants were asked to share how positive or negative they would perceive the feedback statement above if they received it in a social situation. The variable was measured during the second online survey (t_3).

English version: "Thank you! If more people thought this way, the world would be a better place!", own translation.

Participants were asked to share their perception of the above given statement. The variable was measured during the second online survey (t_3).

t3_eval04					
**Values**	**Label**	**Missing**	**Freq.**	**Percent**	**Valid Percent**
1	very negative		1	0.20	0.50
2	negative		6	1.20	2.97
3	neutral		10	2.00	4.95
4	positive		43	8.58	21.29
5	very positive		142	28.34	70.30
not answered		M	299	56.68	
	Sum		501	100.00	100.00
	Valid Cases		202		

t3_eval04					
**Mean**	**Median**	**SD**	**Min**	**Max**	***N***
–	5	–	1	5	202

t3_eval05

T3 – Evaluation FS5: Ich bin absolut der gleichen Meinung! Als denkender und fühlender Mensch muss man so antworten.

Participants were asked to share how positive or negative they would perceive the feedback statement above if they received it in a social situation. The variable was measured during the second online survey (t_3).

English version: “I totally agree! There is no other way to respond for a thinking and feeling human being!”, own translation.

Participants were asked to share their perception of the above given statement. The variable was measured during the second online survey (t_3).

t3_eval05					
**Values**	**Label**	**Missing**	**Freq.**	**Percent**	**Valid Percent**
1	very negative		3	0.60	1.49
2	negative		5	1.00	2.48
3	neutral		14	2.79	6.93
4	positive		42	8.38	20.79
5	very positive		138	27.54	66.32
not answered		M	299	56.68	
	Sum		501	100.00	100.00
	Valid Cases		202		

t3_eval05					
**Mean**	**Median**	**SD**	**Min**	**Max**	N
–	5	–	1	5	202

t3_eval06

T3 – Evaluation FS6: Ähnlich würde ich es auch sehen.

Participants were asked to share how positive or negative they would perceive the feedback statement above if they received it in a social situation. The variable was measured during the second online survey (t_3).

English version: "I tend to agree.", own translation.

Participants were asked to share their perception of the above given statement. The variable was measured during the second online survey (t_3).

t3_eval06					
**Values**	**Label**	**Missing**	**Freq.**	**Percent**	**Valid Percent**
1	very negative		0	0	0
2	negative		0	0	0
3	neutral		47	9.38	23.27
4	positive		146	29.14	72.28
5	very positive		9	1.80	4.46
not answered		M	299	59.68	
	Sum		501	100.00	100.00
	Valid Cases		202		

t3_eval06					
**Mean**	**Median**	**SD**	**Min**	**Max**	*N*
–	4	–	3	5	202

t3_eval07

T3 – Evaluation FS7: Das kann ich prinzipiell nur unterstützen.

Participants were asked to share how positive or negative they would perceive the feedback statement above if they received it in a social situation. The variable was measured during the second online survey (t_3).

English version: "In principle, I agree.", own translation.

Participants were asked to share their perception of the above given statement. The variable was measured during the second online survey (t_3).

t3_eval07					
**Values**	**Label**	**Missing**	**Freq.**	**Percent**	**Valid Percent**
1	very negative		1	0.20	0.50
2	negative		1	0.20	0.50
3	neutral		13	2.59	6.44
4	positive		144	28.74	71.29
5	very positive		43	8.58	21.29
not answered		M	299	59.68	
	Sum		501	100.00	100.00
	Valid Cases		202		

t3_eval07					
**Mean**	**Median**	**SD**	**Min**	**Max**	N
–	4	–	1	5	202

t3_eval08

T3 – Evaluation FS8: Eine grundsätzlich vernünftige Einstellung.

Participants were asked to share how positive or negative they would perceive the feedback statement above if they received it in a social situation. The variable was measured during the second online survey (t_3).

English version: “A generally reasonable attitude.”, own translation.

Participants were asked to share their perception of the above given statement. The variable was measured during the second online survey (t_3).

t3_eval08					
**Values**	**Label**	**Missing**	**Freq.**	**Percent**	**Valid Percent**
1	very negative		0	0.00	0.00
2	negative		1	0.50	0.20
3	neutral		31	15.35	6.19
4	positive		150	74.26	29.94
5	very positive		20	9.90	3.99
not answered		M	299	59.68	
	Sum		501	100.00	100.00
	Valid Cases		202		

t3_eval08					
**Mean**	**Median**	**SD**	**Min**	**Max**	***N***
–	11	–	0	153	202

t3_eval09

T3 – Evaluation FS9: Da kann man nichts dagegen sagen. Ich sehe es eigentlich genauso .

Participants were asked to share how positive or negative they would perceive the feedback statement above if they received it in a social situation. The variable was measured during the second online survey (t_3).

English version: “There is no arguing this. In principle, I agree.”, own translation.

Participants were asked to share their perception of the above given statement. The variable was measured during the second online survey (t_3).

t3_eval09					
**Values**	**Label**	**Missing**	**Freq.**	**Percent**	**Valid Percent**
1	very negative		0	0.00	0.00
2	negative		1	0.20	0.50
3	neutral		34	6.79	16.38
4	positive		123	24.55	60.89
5	very positive		44	8.78	20.91
not answered		M	299	59.68	
	Sum		501	100.00	100.00
	Valid Cases		202		

t3_eval09					
**Mean**	**Median**	**SD**	**Min**	**Max**	N
–	4	–	2	5	202

t3_eval10

T3 – Evaluation FS10: Nach kurzer Überlegung würde ich mich dieser Meinung anschließen.

Participants were asked to share how positive or negative they would perceive the feedback statement above if they received it in a social situation. The variable was measured during the second online survey (t_3).

English version: "After reconsidering it briefly, I agree.", own translation.

Participants were asked to share their perception of the above given statement. The variable was measured during the second online survey (t_3).

t3_eval10					
**Values**	**Label**	**Missing**	**Freq.**	**Percent**	**Valid Percent**
1	very negative		0	0.00	0.50
2	negative		1	0.20	15.35
3	neutral		31	6.19	72.77
4	positive		147	29.34	11.39
5	very positive		23	4.59	10.91
not answered		M	299	59.68	
	Sum		501	100.00	100.00
	Valid Cases		202		

t3_eval10					
**Mean**	**Median**	**SD**	**Min**	**Max**	N
–	4	–	2	5	202

t3_eval11

T3 – Evaluation FS11: Da würde ich nicht mitgehen. Es scheint, als ginge das Denken tendenziell in die falsche Richtung.

Participants were asked to share how positive or negative they would perceive the feedback statement above if they received it in a social situation. The variable was measured during the second online survey (t_3).

English version: “I do not agree. It seems that this kind of reasoning is leading in the wrong direction.”, own translation.

Participants were asked to share their perception of the above given statement. The variable was measured during the second online survey (t_3).

t3_eval11					
**Values**	**Label**	**Missing**	**Freq.**	**Percent**	**Valid Percent**
1	very negative		32	6.39	15.84
2	negative		136	27.15	67.33
3	neutral		23	4.59	11.39
4	positive		10	2.00	4.95
5	very positive		1	0.20	0.50
not answered		M	299	59.68	
	Sum		501	100.00	100.00
	Valid Cases		202		

t3_eval11					
**Mean**	**Median**	**SD**	**Min**	**Max**	N
–	2	–	1	5	202

t3_eval12

T3 – Evaluation FS12: Das ist eine unerwartete, wenig unterstützenswerte Einstellung.

Participants were asked to share how positive or negative they would perceive the feedback statement above if they received it in a social situation. The variable was measured during the second online survey (t_3).

English version: “This attitude is unexpected and hardly worth supporting.”, own translation.

Participants were asked to share their perception of the above given statement. The variable was measured during the second online survey (t_3).

t3_eval12					
**Values**	**Label**	**Missing**	**Freq.**	**Percent**	**Valid Percent**
1	very negative		41	8.18	20.30
2	negative		139	27.74	68.81
3	neutral		11	2.20	5.45
4	positive		8	1.60	3.96
5	very positive		3	0.60	1.49
not answered		M	299	59.68	
	Sum		501	100.00	100.00
	Valid Cases		202		

t3_eval12					
**Mean**	**Median**	**SD**	**Min**	**Max**	N
–	2	–	1	5	202

t3_eval13

T3 – Evaluation FS13: Naja, also mir kommt diese Meinung etwas unüberlegt vor. Ich würde da nicht mitgehen.

Participants were asked to share how positive or negative they would perceive the feedback statement above if they received it in a social situation. The variable was measured during the second online survey (t_3).

English version: "Well, this opinion seems a little inconsiderate to me. I would not go along with it.", own translation.

Participants were asked to share their perception of the above given statement. The variable was measured during the second online survey (t_3).

t3_eval13					
**Values**	**Label**	**Missing**	**Freq.**	**Percent**	**Valid Percent**
1	very negative		8	1.60	3.96
2	negative		146	29.14	72.28
3	neutral		32	6.39	15.84
4	positive		15	2.99	7.43
5	very positive		1	0.20	0.50
not answered		M	299	59.68	
	Sum		497	100.00	100.00
	Valid Cases		220		

t3_eval13					
**Mean**	**Median**	**SD**	**Min**	**Max**	***N***
–	2	–	1	5	202

t3_eval14

T3 – Evaluation FS14: Puh, also das sehe ich anders. Ich finde, dass da nochmal drüber nachgedacht werden sollte

Participants were asked to share how positive or negative they would perceive the feedback statement above if they received it in a social situation. The variable was measured during the second online survey (t_3).

English version: "Phew, well I do not agree with this. I think that this needs to be given more thought.", own translation.

Participants were asked to share their perception of the above given statement. The variable was measured during the second online survey (t_3).

t3_eval14					
**Values**	**Label**	**Missing**	**Freq.**	**Percent**	**Valid Percent**
1	very negative		15	2.99	7.43
2	negative		114	22.75	56.44
3	neutral		47	9.38	23.27
4	positive		25	4.99	12.38
5	very positive		1	0.20	0.50
not answered		M	299	59.68	
	Sum		501	100.00	100.00
	Valid Cases		202		

t3_eval14					
**Mean**	**Median**	**SD**	**Min**	**Max**	N
–	2	–	1	5	202

t3_eval15

T3 – Evaluation FS15: Ich weiß nicht, da hätte ich jetzt schon irgendwie eine andere Einstellung erwartet

Participants were asked to share how positive or negative they would perceive the feedback statement above if they received it in a social situation. The variable was measured during the second online survey (t_3).

English version: "I don't know, I kind of expected a different attitude.", own translation.

Participants were asked to share their perception of the above given statement. The variable was measured during the second online survey (t_3).

t3_eval15					
**Values**	**Label**	**Missing**	**Freq.**	**Percent**	**Valid Percent**
1	very negative		5	1.00	2.48
2	negative		130	25.95	64.36
3	neutral		61	12.18	30.20
4	positive		4	0.80	1.98
5	very positive		2	0.40	0.99
not answered		M	299	59.68	
	Sum		501	100.00	100.00
	Valid Cases		202		

t3_eval15					
**Mean**	**Median**	**SD**	**Min**	**Max**	N
–	2	–	1	5	202

t3_eval16

T3 – Evaluation FS16: Ich frage mich ernsthaft, wie man heutzutage noch so eine Meinung vertreten kann

Participants were asked to share how positive or negative they would perceive the feedback statement above if they received it in a social situation. The variable was measured during the second online survey (t_3).

English version: “Honestly, I find it hard to understand how anyone nowadays could express this kind of opinion!”, own translation.

Participants were asked to share their perception of the above given statement. The variable was measured during the second online survey (t_3).

t3_eval16					
**Values**	**Label**	**Missing**	**Freq.**	**Percent**	**Valid Percent**
1	very negative		141	28.14	69.80
2	negative		47	9.38	23.27
3	neutral		7	1.40	3.47
4	positive		4	0.80	1.98
5	very positive		3	0.60	1.49
not answered		M	299	59.68	
	Sum		501	100.00	100.00
	Valid Cases		202		

t3_eval16					
**Mean**	**Median**	**SD**	**Min**	**Max**	N
–	1	–	1	5	202

t3_eval17

T3 – Evaluation FS17: Au weja, wenn jeder so eine Einstellung hätte, dann wäre echt alles verloren.

Participants were asked to share how positive or negative they would perceive the feedback statement above if they received it in a social situation. The variable was measured during the second online survey (t_3).

English version: “Whew, if everyone shared this kind of attitude, all would be lost!”, own translation.

Participants were asked to share their perception of the above given statement. The variable was measured during the second online survey (t_3).

t3_eval17					
**Values**	**Label**	**Missing**	**Freq.**	**Percent**	**Valid Percent**
1	very negative		167	33.33	82.67
2	negative		27	5.39	13.37
3	neutral		6	1.20	2.97
4	positive		2	0.40	0.99
5	very positive		0	0.00	0.00
not answered		M	299	59.68	
	Sum		501	100.00	100.00
	Valid Cases		202		

t3_eval17					
**Mean**	**Median**	**SD**	**Min**	**Max**	N
–	1	–	1	4	202

t3_eval18

T3 – Evaluation FS18: Autsch, das tut weh! Wie kommt man dazu, so über das Thema zu denken?

Participants were asked to share how positive or negative they would perceive the feedback statement above if they received it in a social situation. The variable was measured during the second online survey (t_3).

English version: "Ouch, that hurts! How could anyone start thinking this way about the topic?", own translation.

Participants were asked to share their perception of the above given statement. The variable was measured during the second online survey (t_3).

t3_eval18					
**Values**	**Label**	**Missing**	**Freq.**	**Percent**	**Valid Percent**
1	very negative		149	29.74	73.76
2	negative		47	9.38	23.27
3	neutral		6	1.20	2.97
4	positive		0	0.20	0.00
5	very positive		0	0.00	0.00
not answered		M	299	59.68	
	Sum		501	100.00	100.00
	Valid Cases		202		

t3_eval18					
**Mean**	**Median**	**SD**	**Min**	**Max**	***N***
–	1	–	1	3	202

t3_eval19

T3 – Evaluation FS19: Eine solche Meinung verstößt gegen jede gesellschaftlich akzeptable Vorstellung vom Guten und Richtigen!

Participants were asked to share how positive or negative they would perceive the feedback statement above if they received it in a social situation. The variable was measured during the second online survey (t_3).

English version: “This kind of opinion contradicts every socially accepted notion of what is right and good!”, own translation.

Participants were asked to share their perception of the above given statement. The variable was measured during the second online survey (t_3).

t3_eval19					
**Values**	**Label**	**Missing**	**Freq.**	**Percent**	**Valid Percent**
1	very negative		150	29.94	74.26
2	negative		39	7.78	19.31
3	neutral		9	1.80	4.46
4	positive		2	0.40	0.99
5	very positive		2	0.40	0.99
not answered		M	299	59.68	
	Sum		501	100.00	100.00
	Valid Cases		202		

t3_eval19					
**Mean**	**Median**	**SD**	**Min**	**Max**	N
–	1	–	1	5	202

t3_eval20

T3 – Evaluation FS20: Furchtbare Einstellung! Lehne ich entschieden ab!

Participants were asked to share how positive or negative they would perceive the feedback statement above if they received it in a social situation. The variable was measured during the second online survey (t_3).

English Version: "Horrible attitude! I strictly object!", own translation.

Participants were asked to share their perception of the above given statement on a scale ranging from very negative to very positive. The variable was measured during the second online survey (t_3).

t3_eval20					
**Values**	**Label**	**Missing**	**Freq.**	**Percent**	**Valid Percent**
1	very negative		171	34.13	84.65
2	negative		23	4.59	11.39
3	neutral		4	0.80	1.98
4	positive		3	0.60	1.49
5	very positive		1	0.20	0.50
not answered		M	299	59.68	
	Sum		501	100.00	100.00
	Valid Cases		202		

t3_eval20					
**Mean**	**Median**	**SD**	**Min**	**Max**	***N***
–	1	–	1	5	202

t3_lastdata

T3 – Time when the dataset was last changed.

System variable that was automatically generated by SoSci Survey (t_3).

t3_lastdata					
**Values**	**Label**	**Missing**	**Freq.**	**Percent**	**Valid Percent**
24/10/18			94	18.76	34.81
25/10/18			37	7.39	13.70
26/10/18			13	2.59	4.81
27/10/18			60	11.98	22.22
28/10/18			28	5.59	10.37
29/10/18			16	3.19	5.93
30/10/18			11	2.20	4.07
31/10/18			7	1.40	2.59
1/11/18			2	0.40	0.74
2/11/18			2	0.40	0.74
not answered		M	231	46.11	
	Sum		501	100.00	100.00
	Valid Cases		270		

t3_lastpage

T3 – Page the participants last edited

System variable that was automatically generated by SoSci Survey (t_3).

t3_lastpage					
**Values**	**Label**	**Missing**	**Freq.**	**Percent**	**Valid Percent**
2			1	0.20	0.37
3			3	0.60	1.11
8			1	0.20	0.37
11			1	0.20	0.37
12			264	52.69	97.78
not answered		M	231	46.11	
	Sum		501	100.00	100.00
	Valid Cases		270		

t3_quest

T3 – Questionnaire version

System variable that was automatically generated by SoSci Survey (t_3). It indicates which questionnaire was presented to the participants.

t3_quest					
**Values**	**Label**	**Missing**	**Freq.**	**Percent**	**Valid Percent**
0	control group		64	12.77	23.70
1	treatment group(s)		206	41.12	76.30
not answered		M	231	46.11	
	Sum		501	100.00	100.00
	Valid Cases		270		

t3_sp

T3 – Speciesism Index

Index variable measuring participants' overall score on the Speciesism Scale. The variables involved in the formation were measured on a scale from 0 to 100 during the first online survey (t_3).

t3_sp					
**Values**	**Label**	**Missing**	**Freq.**	**Percent**	**Valid Percent**
valid values			255	50.90	100.00
not answered		M	246	49.10	
	Sum		501	100.00	100.00
	Valid Cases		255		

t3_sp (valid values only)					
**Mean**	**Median**	**SD**	**Min**	**Max**	***N***
36.21	34.91	18.18	0	77	255

Windrich, Ivo. 2017. "Zur Messung speziesistischer Einstellungen". Leipzig, *Arbeitsbericht des Instituts für Soziologie*, No. 72.

t3_sp01

T3 – Speciesism: Ich finde es in Ordnung, dass Tiere für die Fleischproduktion getötet werden.

English version: "I agree with the killing of animals for meat production.", own translation.

Speciesism Scale item. The variable was measured on a scale from 0 to 100 during the first online survey (t_3).

t3_sp01					
**Values**	**Label**	**Missing**	**Freq.**	**Percent**	**Valid Percent**
valid values			263	52.50	100.00
I don't know		M	3	0.60	
not answered		M	235	46.91	
	Sum		501	100.00	100.00
	Valid Cases		263		

t3_sp01 (valid values only)					
**Mean**	**Median**	**SD**	**Min**	**Max**	***N***
42.71	43	32.69	0	100	263

Windrich, Ivo. 2017. "Zur Messung speziesistischer Einstellungen". Leipzig, *Arbeitsbericht des Instituts für Soziologie*, No. 72.

t3_sp02

T3 – Speciesism: Tiere sind zum Nutzen des Menschen da.

English version: "Animals exist for the benefit of humans.", own translation.

Speciesism Scale item. The variable was measured on a scale from 0 to 100 during the second online survey (t_3).

t3_sp02					
**Values**	**Label**	**Missing**	**Freq.**	**Percent**	**Valid Percent**
valid values			262	52.30	100.00
I don't know		M	4	0.80	
not answered		M	235	46.91	
	Sum		501	100.00	100.00
	Valid Cases		262		

t3_sp02 (valid values only)					
**Mean**	**Median**	**SD**	**Min**	**Max**	N
28.21	20	27.18	0	100	262

Windrich, Ivo. 2017. "Zur Messung speziesistischer Einstellungen". Leipzig, *Arbeitsbericht des Instituts für Soziologie*, No. 72.

t3_sp03

T3–Speciesism: Ich lehne Tierversuche ab (reverse wording).

English version: "I oppose animal testing.", own translation.

Speciesism Scale item. The variable was measured on a scale from 0 to 100 during the second online survey (t_3).

t3_sp03					
**Values**	**Label**	**Missing**	**Freq.**	**Percent**	**Valid Percent**
valid values			266	53.09	100.00
not answered		M	235	46.91	
	Sum		501	100.00	100.00
	Valid Cases		266		

t3_sp03 (valid values only)					
**Mean**	**Median**	**SD**	**Min**	**Max**	*N*
34.88	23.5	33.52	0	100	266

Remarks: The item was reverse worded and has been recoded accordingly.

Windrich, Ivo. 2017. "Zur Messung speziesistischer Einstellungen". Leipzig, *Arbeitsbericht des Instituts für Soziologie*, No. 72.

t3_sp04

T3 – Speciesism: Nur Menschen haben ein Bewusstsein, Tiere haben kein Bewusstsein.

English version: “Only human beings have consciousness, animals do not have consciousness.”, own translation.

Speciesism Scale item. The variable was measured on a scale from 0 to 100 during the second online survey (t_3).

t3_sp04					
**Values**	**Label**	**Missing**	**Freq.**	**Percent**	**Valid Percent**
valid values			258	51.50	100.00
I don't know		M	8	1.60	
not answered		M	235	46.91	
	Sum		501	100.00	100.00
	Valid Cases		258		

t3_sp04 (valid values only)					
**Mean**	**Median**	**SD**	**Min**	**Max**	***N***
15.60	6.5	21.12	0	100	258

Windrich, Ivo. 2017. “Zur Messung speziesistischer Einstellungen”. Leipzig, *Arbeitsbericht des Instituts für Soziologie*, No. 72.

t3_sp05

T3 – Speciesism: Die Vernunft trennt den Menschen vom Tier.

English version: "Reason separates human beings from animals.", own translation.

Speciesism Scale item. The variable was measured on a scale from 0 to 100 during the second online survey (t_3).

t3_sp05					
**Values**	**Label**	**Missing**	**Freq.**	**Percent**	**Valid Percent**
valid values			263	52.50	100.00
I don't know		M	3	0.60	
not answered		M	235	46.91	
	Sum		501	100.00	100.00
	Valid Cases		263		

t3_sp05 (valid values only)					
**Mean**	**Median**	**SD**	**Min**	**Max**	***N***
53.67	55	31.04	0	100	263

Windrich, Ivo. 2017. "Zur Messung speziesistischer Einstellungen". Leipzig, *Arbeitsbericht des Instituts für Soziologie*, No. 72.

t3_sp06

T1 – Speciesism: Die Massentierhaltung ist nicht mit den Moralvorstellungen unserer Gesellschaft vereinbar (reverse wording)

English version: "Intensive livestock farming is incompatible with our society's code of ethics.", own translation.

Attention: This variable is not part of the original Speciesism Scale.

The variable was measured on a scale from 0 to 100 during the second online survey (t_3).

t3_sp06					
**Values**	**Label**	**Missing**	**Freq.**	**Percent**	**Valid Percent**
valid values			266	53.09	100.00
not answered		M	235	46.91	
	Sum		501	100.00	100.00
	Valid Cases		266		

t3_sp06 (valid values only)					
**Mean**	**Median**	**SD**	**Min**	**Max**	*N*
27.68	19	29.23	0	100	266

Remarks: The item was reverse worded and has been recoded accordingly.

t3_sp07

T3 – Speciesism: Die Menschen sollten Tiere als fühlende Mitlebewesen respektieren (reverse wording).

English version: “Humans should respect animals as sentient co-beings.”, own translation.

Speciesism Scale item. The variable was measured on a scale from 0 to 100 during the second online survey (t_3).

t3_sp07					
**Values**	**Label**	**Missing**	**Freq.**	**Percent**	**Valid Percent**
valid values			266	53.09	100.00
not answered		M	235	46.91	
	Sum		501	100.00	100.00
	Valid Cases		266		

t3_sp07 (valid values only)					
**Mean**	**Median**	**SD**	**Min**	**Max**	***N***
16.05	11	17.87	0	91	266

Remarks: The item was reverse worded and has been recoded accordingly.

Windrich, Ivo. 2017. “Zur Messung speziesistischer Einstellungen”. Leipzig, *Arbeitsbericht des Instituts für Soziologie*, No. 72.

t3_sp08

T3 – Speciesism: Tiere sind ihrem Trieb vollkommen unterworfen.

English version: "Animals are completely subdued to their instincts.", own translation.

Speciesism Scale item. The variable was measured on a scale from 0 to 100 during the second online survey (t_3).

t3_sp08					
**Values**	**Label**	**Missing**	**Freq.**	**Percent**	**Valid Percent**
valid values			266	53.09	100.00
not answered		M	235	46.91	
	Sum		501	100.00	100.00
	Valid Cases		266		

t3_sp08 (valid values only)					
**Mean**	**Median**	**SD**	**Min**	**Max**	***N***
49.01	49	27.54	0	100	266

Windrich, Ivo. 2017. "Zur Messung speziesistischer Einstellungen". Leipzig, *Arbeitsbericht des Instituts für Soziologie*, No. 72.

t3_sp09

T3 – Speciesism: Tiere zu töten und zu essen gehört einfach zum Wesen des Menschen.

English version: "The killing and eating of animals is part of human nature.", own translation.

Speciesism Scale item. The variable was measured on a scale from 0 to 100 during the second online survey (t_3).

t3_sp09					
**Values**	**Label**	**Missing**	**Freq.**	**Percent**	**Valid Percent**
valid values			264	52.69	100.00
I don't know		M	2	0.40	
not answered		M	235	46.91	
	Sum		501	100.00	100.00
	Valid Cases		264		

t3_sp09 (valid values only)					
**Mean**	**Median**	**SD**	**Min**	**Max**	N
41.84	35.5	34.15	0	100	264

Windrich, Ivo. 2017. "Zur Messung speziesistischer Einstellungen". Leipzig, *Arbeitsbericht des Instituts für Soziologie*, No. 72.

t3_sp10

T3 – Speciesism: Ich betrachte Tiere als gleichwertig zum Menschen (reverse wording).

English version: “I consider animals to be of equal value as human beings.”, own translation.

Speciesism Scale item. The variable was measured on a scale from 0 to 100 during the second online survey (t_3).

t3_sp10					
**Values**	**Label**	**Missing**	**Freq.**	**Percent**	**Valid Percent**
valid values			266	53.09	100.00
not answered		M	235	46.91	
	Sum		501	100.00	100.00
	Valid Cases		266		

t3_sp10 (valid values only)					
**Mean**	**Median**	**SD**	**Min**	**Max**	*N*
48.7	51	30.47	0	100	266

Remarks: The item was reverse worded and has been recoded accordingly.

Windrich, Ivo. 2017. “Zur Messung speziesistischer Einstellungen”. Leipzig, *Arbeitsbericht des Instituts für Soziologie*, No. 72.

t3_sp11

T3 – Speciesism: Der Mensch soll bestimmen, was mit Tieren passiert.

English version: “Human beings should decide over animals.”, own translation.

Speciesism Scale item. The variable was measured on a scale from 0 to 100 during the second online survey (t_3).

t3_sp11					
**Values**	**Label**	**Missing**	**Freq.**	**Percent**	**Valid Percent**
valid values			262	52.30	100.00
I don't know		M	4	0.80	
not answered		M	235	46.91	
	Sum		501	100.00	100.00
	Valid Cases		262		

t3_sp11 (valid values only)					
**Mean**	**Median**	**SD**	**Min**	**Max**	*N*
27.94	21.5	25.58	0	100	262

Windrich, Ivo. 2017. “Zur Messung speziesistischer Einstellungen”. Leipzig, *Arbeitsbericht des Instituts für Soziologie*, No. 72.

t3_sp12

T3 – Speciesism: Fische können Schmerzen empfinden. (reverse wording)

English version: "Fish can feel pain.", own translation.

Speciesism Scale item. The variable was measured on a scale from 0 to 100 during the second online survey (t_3).

t3_sp12					
**Values**	**Label**	**Missing**	**Freq.**	**Percent**	**Valid Percent**
valid values			266	53.09	100.00
not answered		M	235	46.91	
	Sum		501	100.00	100.00
	Valid Cases		266		

t3_sp12 (valid values only)					
**Mean**	**Median**	**SD**	**Min**	**Max**	N
22.64	17.5	24.29	0	100	266

Remarks: The item was reverse worded and has been recoded accordingly.

Windrich, Ivo. 2017. "Zur Messung speziesistischer Einstellungen". Leipzig, *Arbeitsbericht des Instituts für Soziologie*, No. 72.

t3_sp13

T3 – Speciesism: Meiner Meinung nach steht der Mensch über dem Tier.

English version: "In my opinion human beings are superior to animals.", own translation.

Speciesism Scale item. The variable was measured on a scale from 0 to 100 during the second online survey (t_3).

t3_sp13					
**Values**	**Label**	**Missing**	**Freq.**	**Percent**	**Valid Percent**
valid values			264	52.69	100.00
I don't know		M	2	0.40	
not answered		M	235	46.91	
	Sum		501	100.00	100.00
	Valid Cases		264		

t3_sp13 (valid values only)					
**Mean**	**Median**	**SD**	**Min**	**Max**	N
46.92	51	33.57	0	100	264

Windrich, Ivo. 2017. "Zur Messung speziesistischer Einstellungen". Leipzig, *Arbeitsbericht des Instituts für Soziologie*, No. 72.

t3_sp14

T3 – Speciesism: Nutztiere sind nun einmal dazu da geschlachtet zu werden.

English version: "Livestock exists to be slaughtered.", own translation.

Speciesism Scale item. The variable was measured on a scale from 0 to 100 during the second online survey (t_3).

t3_sp14					
**Values**	**Label**	**Missing**	**Freq.**	**Percent**	**Valid Percent**
valid values			265	52.89	100.00
I don't know		M	1	0.20	
not answered		M	235	46.91	
	Sum		501	100.00	100.00
	Valid Cases		265		

t3_sp14 (valid values only)					
**Mean**	**Median**	**SD**	**Min**	**Max**	N
35.21	27	32.07	0	100	265

Windrich, Ivo. 2017. "Zur Messung speziesistischer Einstellungen". Leipzig, *Arbeitsbericht des Instituts für Soziologie*, No. 72.

t3_sp15

T3 – Speciesism: Ich rege mich manchmal auf, wenn ich wilde Tiere in Käfigen im Zoo sehe (reverse wording).

English version: “I sometimes get furious upon viewing wild animals caged in zoos.”, own translation.

Speciesism Scale item. The variable was measured on a scale from 0 to 100 during the second online survey (t_3).

t3_sp15					
**Values**	**Label**	**Missing**	**Freq.**	**Percent**	**Valid Percent**
valid values			266	53.09	100.00
not answered		M	235	46.91	
	Sum		501	100.00	100.00
	Valid Cases		266		

t3_sp15 (valid values only)					
**Mean**	**Median**	**SD**	**Min**	**Max**	***N***
31.98	25.5	28.17	0	100	266

Remarks: The item was reverse worded and has been recoded accordingly.

Windrich, Ivo. 2017. “Zur Messung speziesistischer Einstellungen”. Leipzig, *Arbeitsbericht des Instituts für Soziologie*, No. 72.

t3_sp16

T3 – Speciesism: Manche Tiere sind Nahrungsmittel.

English version: “Some animals are food items.”, own translation.

Speciesism Scale item. The variable was measured on a scale from 0 to 100 during the second online survey (t_3).

t3_sp16					
**Values**	**Label**	**Missing**	**Freq.**	**Percent**	**Valid Percent**
valid values			265	56.94	100.00
I don't know		M	1	0.20	
not answered		M	235	46.91	
	Sum		501	100.00	100.00
	Valid Cases		265		

t3_sp16 (valid values only)					
**Mean**	**Median**	**SD**	**Min**	**Max**	***N***
42.54	43	34.72	0	100	265

Windrich, Ivo. 2017. “Zur Messung speziesistischer Einstellungen”. Leipzig, *Arbeitsbericht des Instituts für Soziologie*, No. 72.

t3_sp17

T3 – Speciesism: Manche Tiere können Leid, Freude und Trauer empfinden (reverse wording).

English version: “Some animals are capable of feeling pain, joy and grief.”, own translation.

Speciesism Scale item. The variable was measured on a scale from 0 to 100 during the second online survey (t_3).

t3_sp17					
**Values**	**Label**	**Missing**	**Freq.**	**Percent**	**Valid Percent**
valid values			266	53.09	100.00
not answered		M	235	46.91	
	Sum		501	100.00	100.00
	Valid Cases		266		

t3_sp17 (valid values only)					
**Mean**	**Median**	**SD**	**Min**	**Max**	***N***
9.66	1	15.56	0	100	266

Remarks: The item was reverse worded and has been recoded accordingly.

Windrich, Ivo. 2017. “Zur Messung speziesistischer Einstellungen”. Leipzig, *Arbeitsbericht des Instituts für Soziologie*, No. 72.

t3_sp18

T3 – Speciesism: Wenn Kühe ein glückliches Leben haben, dann ist es in Ordnung ihre Milch zu nehmen.

English version: "If cows live a happy life it is acceptable to take their milk.", own translation.

Speciesism Scale item. The variable was measured on a scale from 0 to 100 during the second online survey (t_3).

t3_sp18					
**Values**	**Label**	**Missing**	**Freq.**	**Percent**	**Valid Percent**
valid values			265	52.89	100.00
I don't know		M	1	0.20	
not answered		M	235	46.91	
	Sum		501	100.00	100.00
	Valid Cases		265		

t3_sp18 (valid values only)					
**Mean**	**Median**	**SD**	**Min**	**Max**	N
66.84	72	29.90	0	100	265

Windrich, Ivo. 2017. "Zur Messung speziesistischer Einstellungen". Leipzig, *Arbeitsbericht des Instituts für Soziologie*, No. 72.

t3_sp19

T3 – Speciesism: Der Körper eines Schweines gehört ausschließlich dem Schwein (reverse wording).

English version: “A pig’s body belongs exclusively to the pig.”, own translation.

Speciesism Scale item. The variable was measured on a scale from 0 to 100 during the second online survey (t_3).

t3_sp19					
**Values**	**Label**	**Missing**	**Freq.**	**Percent**	**Valid Percent**
valid values			266	53.09	100.00
not answered		M	235	46.91	
	Sum		501	100.00	100.00
	Valid Cases		266		

t3_sp19 (valid values only)					
**Mean**	**Median**	**SD**	**Min**	**Max**	***N***
33.99	30.5	29.74	0	100	266

Remarks: The item was reverse worded and has been recoded accordingly.

Windrich, Ivo. 2017. “Zur Messung speziesistischer Einstellungen”. Leipzig, *Arbeitsbericht des Instituts für Soziologie*, No. 72.

t3_sp20

T3 – Speciesism: Tiere sollten ihr eigenes Leben leben dürfen (reverse wording).

English version: “Animals should be allowed to live their own life.”, own translation.

Speciesism Scale item. The variable was measured on a scale from 0 to 100 during the second online survey (t_3).

t3_sp20					
**Values**	**Label**	**Missing**	**Freq.**	**Percent**	**Valid Percent**
valid values			266	53.09	100.00
not answered		M	235	46.91	
	Sum		501	100.00	100.00
	Valid Cases		266		

t3_sp20 (valid values only)					
**Mean**	**Median**	**SD**	**Min**	**Max**	***N***
27.79	27	23.59	0	98	266

Remarks: The item was reverse worded and has been recoded accordingly.

Windrich, Ivo. 2017. “Zur Messung speziesistischer Einstellungen”. Leipzig, *Arbeitsbericht des Instituts für Soziologie*, No. 72.

t3_sp21

T3 – Speciesism: Das Nutztiere getötet werden, ist kein besonderes Problem, es sind ja bloß Tiere.

English version: "Killing of livestock is not problematic, it's just animals.", own translation.

Speciesism Scale item. The variable was measured on a scale from 0 to 100 during the second online survey (t_3).

t3_sp21					
**Values**	**Label**	**Missing**	**Freq.**	**Percent**	**Valid Percent**
valid values			265	52.89	100.00
I don't know		M	1	0.20	
not answered		M	235	46.91	
	Sum		501	100.00	100.00
	Valid Cases		265		

t3_sp21 (valid values only)					
**Mean**	**Median**	**SD**	**Min**	**Max**	N
21.92	11	25.34	0	100	265

Windrich, Ivo. 2017. "Zur Messung speziesistischer Einstellungen". Leipzig, *Arbeitsbericht des Instituts für Soziologie*, No. 72.

t3_sp22

T3 – Speciesism: Ich finde, dass das Schlachten von Tieren ein Ende finden sollte (reverse wording).

English version: "I think there should be an end to slaughtering animals.", own translation.

Speciesism Scale item. The variable was measured on a scale from 0 to 100 during the second online survey (t_3).

t3_sp22					
**Values**	**Label**	**Missing**	**Freq.**	**Percent**	**Valid Percent**
valid values			266	53.09	100.00
not answered		M	235	46.91	
	Sum		501	100.00	100.00
	Valid Cases		266		

t3_sp22 (valid values only)					
**Mean**	**Median**	**SD**	**Min**	**Max**	***N***
47.62	48.5	33.99	0	100	266

Remarks: The item was reverse worded and has been recoded accordingly.

Windrich, Ivo. 2017. "Zur Messung speziesistischer Einstellungen". Leipzig, *Arbeitsbericht des Instituts für Soziologie*, No. 72.

t3_sp23

T3 – Speciesism: Wenn ich einen Tiertransporter sehe, dann macht mich das wütend oder traurig (reverse wording).

English version: "Whenever I see an animal transport I get angry or sad.", own translation.

Speciesism Scale item. The variable was measured on a scale from 0 to 100 during the second online survey (t_3).

t3_sp23					
**Values**	**Label**	**Missing**	**Freq.**	**Percent**	**Valid Percent**
valid values			266	53.09	100.00
not answered		M	235	46.91	
	Sum		501	100.00	100.00
	Valid Cases		266		

t3_sp23 (valid values only)					
**Mean**	**Median**	**SD**	**Min**	**Max**	***N***
35.02	28.5	30.58	0	100	266

Windrich, Ivo. 2017. "Zur Messung speziesistischer Einstellungen". Leipzig, *Arbeitsbericht des Instituts für Soziologie*, No. 72.

t3_sp24

T3 – Speciesism: Der Schmerz eines Menschen zählt mehr als der Schmerz einer Maus.

English version: "The pain of a human being is of greater relevance than a mouse's pain.", own translation.

Speciesism Scale item. The variable was measured on a scale from 0 to 100 during the second online survey (t_3).

t3_sp24					
**Values**	**Label**	**Missing**	**Freq.**	**Percent**	**Valid Percent**
valid values			263	52.50	100.00
I don't know		M	3	0.60	
not answered		M	235	46.91	
	Sum		501	100.00	100.00
	Valid Cases		263		

t3_sp24 (valid values only)					
**Mean**	**Median**	**SD**	**Min**	**Max**	***N***
47.34	51	33.05	0	100	263

Windrich, Ivo. 2017. "Zur Messung speziesistischer Einstellungen". Leipzig, *Arbeitsbericht des Instituts für Soziologie*, No. 72.

t3_started

T3 – Time at which the interview started (in October/November 2018)

System variable that was automatically generated by SoSci Survey (t_1).

t3_started					
**Values**	**Label**	**Missing**	**Freq.**	**Percent**	**Valid Percent**
24 Oct 18			95	18.96	35.19
25 Oct 18			36	7.19	13.33
26 Oct 18			14	2.79	5.19
27 Oct 18			59	11.78	21.85
28 Oct 18			29	5.79	10.74
29 Oct 18			15	2.99	5.56
30 Oct 18			11	2.20	4.07
31 Oct 18			7	1.40	2.59
01 Nov 18			2	0.40	0.74
02 Nov 18			2	0.40	0.74
not available		M	231	46.11	
	Sum		501	100.00	100.00
	Valid Cases		270		

t3_time_sum

T3 – Total time spent (outliers excluded)

System variable that was automatically generated by SoSci Survey (t_3).

t3_time_sum					
**Mean**	**Median**	**SD**	**Min**	**Max**	*N*
419.58	413	126.96	8	751	270

tg

T3 – Treatment group membership

The variable indicates to which treatment condition participants were randomly assigned.

t3_tg					
**Values**	**Label**	**Missing**	**Freq.**	**Percent**	**Valid Percent**
control group			58	11.58	21.01
positive-positive			53	10.58	19.20
negative-negative			67	13.37	24.28
positive-negative			50	9.98	18.12
negative-positive			48	9.58	17.39
not available		M	225	44.91	
	Sum		501	100.00	100.00
	Valid Cases		276		
